# Testing Transgenic Aspen Plants with bar Gene for Herbicide Resistance under Semi-natural Conditions

**Published:** 2016

**Authors:** V. G. Lebedev, V. N. Faskhiev, N. P. Kovalenko, K. A. Shestibratov, A. I. Miroshnikov

**Affiliations:** Branch of Shemyakin and Ovchinnikov Institute of Bioorganic Chemistry, Russian Academy of Sciences, Science av., 6, 142290, Pushchino, Russia; Lomonosov Moscow State University, Leninskie Gory, 1/51, 119991, Moscow, Russia

**Keywords:** aspen, transgenic plants, bar gene, phosphinothricin, herbicide resistance

## Abstract

Obtaining herbicide resistant plants is an important task in the genetic engineering of forest trees. Transgenic European aspen plants (*Populus tremula *L.) expressing the *bar *gene for phosphinothricin resistance have been produced using *Agrobacterium tumefaciens*-mediated transformation. Successful genetic transformation was confirmed by PCR analysis for thirteen lines derived from two elite genotypes. In 2014–2015, six lines were evaluated for resistance to herbicide treatment under semi-natural conditions. All selected transgenic lines were resistant to the herbicide Basta at doses equivalent to 10 l/ha (twofold normal field dosage) whereas the control plants died at 2.5 l/ha. Foliar NH4-N concentrations in transgenic plants did not change after treatment. Extremely low temperatures in the third ten-day period of October 2014 revealed differences in freeze tolerance between the lines obtained from Pt of f2 aspen genotypes. Stable expression of the *bar *gene after overwintering outdoors was confirmed by RT-PCR. On the basis of the tests, four transgenic aspen lines were selected. The *bar* gene could be used for retransformation of transgenic forest trees expressing valuable traits, such as increased productivity.

## INTRODUCTION

In the early stages of their growth, many trees (such as willow and poplar) cannot compete with weeds, making weed control essential [1]. This problem is particularly acute in nurseries, where the low competitiveness of young plants reduces their survival rate and weakens their growth. Therefore, nurseries spend 50–70% of the funds allocated to the cultivation of standard planting material on weed control [2]. Mechanical methods are labor-intensive and have low efficiency. Easy-to-use, efficient, and economical herbicides are more promising.

In the temperate zone, forest nurseries grow various types of *Populus*; however, the use of selective herbicides is almost impossible, since poplars are very sensitive to most herbicides [3]. It seems preferable to grow plants that are resistant to highly efficient non-selective herbicides that are relatively safe for the environment.

To this end, various genes conferring resistance to herbicides were inserted into woody plants. The first such gene was *aroA *that confers resistance to glyphosate [4]. There were also reports on a transfer of the* crsl-1 *gene to acquire resistance to sulfonylurea [5], the *CP4 *and *GOX *genes for glyphosate resistance [6]. However, the most frequently used gene is the *bar* gene from soil bacterium *Streptomyces hygroscopicus*, which confers resistance to broad-spectrum herbicides (Liberty, Basta, Finale, etc.) that are based on phosphinothricin (PPT, ammonium glufosinate). PPT is an analogue of *L*-glutamic acid and a potent inhibitor of glutamine synthetase (GS), which plays the central role in ammonium assimilation and regulation of nitrogen metabolism in plants [7]. Inhibition of GS results in rapid accumulation of ammonium in a plant cell and its subsequent death [8]. The *bar *gene encodes the PPT acetyltransferase enzyme which acetylates a free amino group in PPT and thereby inactivates it [9]. The *bar* gene was inserted into different species and hybrids of* Populus* [3, 10] and *Eucalyptus *[11, 12], as well as oak [13] and various coniferous [14, 15]; however, aspen plants have not been transformed. The aim of our work was to create herbicide-resistant aspen plants by transforming Russian highly productive aspen genotypes with the *bar *gene and to use testing under semi-natural conditions to select lines that look promising for plantation forestry.

## MATERIALS AND METHODS

We used aspen plants (*P. tremula *L.) of two genotypes: Pt and f2. Plants of the Pt genotype were discovered in the Leningrad region, and it is characterized by rapid growth and resistance to trunk rot (SPbNIILH, A.V. Zhigunov, personal communication), whereas plants of the f2 genotype represent *in vitro *culture of clone 34, which was discovered by S.N. Bagaev in the Kostroma region [16]. The plants were grown *in vitro *at 22–24 °C on the WPM medium [17] with 0.5 mg/l gibberellin and a photoperiod of 16 hours.

The transformation was performed by the *Agrobacterium tumefaciens *CBE21 strain with a binary pBIBar vector [18] containing the nos-*nptII *and 35S-*bar *genes according to [19]. Kanamycin-resistant transformants were analyzed by PCR. Plant DNA was isolated according to [20]. Potential agrobacterial contamination of DNA preparations was checked by amplification of the* virB *gene sequence. The following primer pairs were used:

Vir-B1 – 5’-GGCTACATCGAAGATCGTATGAATG- 3’;

Vir-B2 – 5’-GACTATAGCGATGGTTACGATGTTGAC- 3’;

Nos – 5’-CGCGGGTTTCTGGAGTTTAATGAGCTAAG- 3’;

NptII – 5’-GCATGCGCGCCTTGAGCCTGG-3’;

Bar-1 – 5’-TGCACCATCGTCAACCACTA-3’;

Bar-2 – 5’-ACAGCGACCACGCTCTTGAA-3’.

The reaction mixture contained 16 mM (NH_4_)_2_SO_4_, 0.01% bovine serum albumin, 200 μM of each dNTP, 0.4 •µM of each oligonucleotide, 0.05 activity units/l Taq-polymerase, and 1– ng/•µl genomic DNA. The PCR conditions: denaturation at 96 °C (3 min); 30 cycles at 94 °C (1 min), 60 °C (*nptII*, *bar*) or 58 °C (*virB*) for 1 min, 72 °C (1 min); elongation at 72 °C (5 min). The reaction was carried out in a MJ MiniTM Gradient Thermal Cycler (Bio-Rad, USA).

The resistance of transgenic lines was assessed *in vitro *by rooting plants in the WPM medium with 0, 0.5 or 5 mg/l PPT. Rate of rooting, number of roots, and their length were assessed two weeks after planting. To evaluate the resistance to herbicide treatment, the transgenic and control plants were micropropagated, acclimatized to greenhouse conditions and following the transplantation into 1L plastic pots with peat:perlite substrate (3:1) were transferred outdoors at the Branch of the Shemyakin and Ovchinnikov Institute of Bioorganic Chemistry RAS in Pushchino in the beginning of June 2014. In mid-July, the plants were treated with water (control) or 0.5, 1 and 2% aqueous solution of herbicide Basta (Bayer CropScience, 150 g/l PPT) in doses equivalent to 2.5, 5, and 10 l/ha (four plants of each line for each treatment). Visual assessment of the damage was performed 3, 7, 14, and 28 days after the treatment using the following scale: 0 points, no damage; 1, 0–25% necrosis of leaf surface; 2, 25–50% necrosis; 3, 50–75% necrosis; 4, 75–100% necrosis; 5, complete necrosis of leaves. On the day of treatment and 3 days after the treatment, the leaves were collected to assess the content of ammonium nitrogen and water. The plant material was extracted according to [21]. Ammonium nitrogen was determined according to [22]. Water content was determined by drying at 105 °C for 24 h. During the 2014 growing season, plant height and number of leaves were measured every four weeks and basal diameter was measured every 8 weeks.

In May 2015, the plants were transplanted into 3L pots after overwintering outdoors. The degree of frost damage to plants was determined by the ratio of the living part to the total stem length. Meteorological data were obtained from the automatic weather station UGT in Puschino (ca. 600 m away from the test area). The expression of the *bar *gene was evaluated in June 2015 by RT-PCR (actin gene was used as the internal control). RNA was isolated by a modified method [23]. cDNA was synthesized in two steps. At the first stage, the reaction mixture (0.1–5 •µg RNA, 0.5 •µg oligo- dT-primer, 10 activity units of the RNase inhibitor) was heated for 5 min at 70 °C and transferred into ice. At the second stage, 0.4 mM dNTP, reverse transcriptase buffer, and 4 activity units/•µl of M-MuLV reverse transcriptase were added to the mixture and it was incubated for 1.5 h at 37°C and then heated (15 min at 70°C). PCR was performed using the following primers for the *bar *gene and actin:

Actin 1 up – 5’-TATGCCCTCCCACATGCCAT-3’;

Actin 1 low – 5’-CATCTGCTGGAAGGTGCTGA-3’.

The reaction mixture contained ScreenMix-HS (“Evrogen”), 0.8 mM primers, 0.1– •µg RNA or cDNA. PCR conditions were the following: denaturation at 95°C (5 min); 31 cycles at 95°C (45 s), 59°C (30 s), 72°C (1 min); and elongation at 72°C (10 min). In July 2015, the plants were treated with herbicide according to the procedure described above.

Statistical processing was performed using the Statistica 6.1 (StatSoft, USA) software.

## RESULTS

**Fig. 1:**
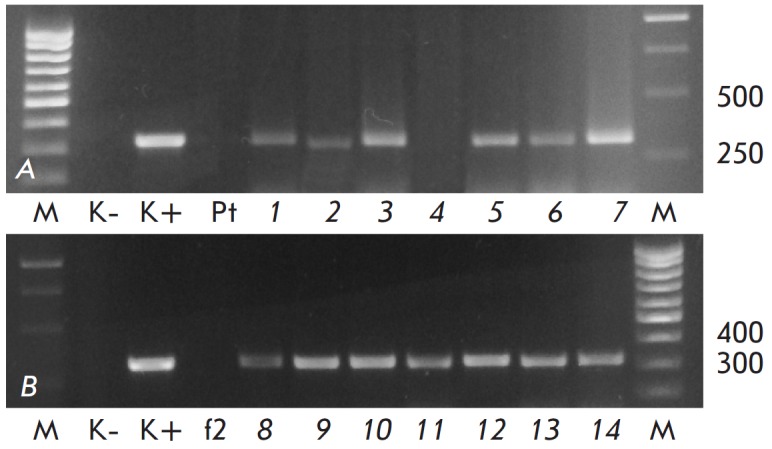
PCR analysis of transgenic aspen plants for integration of the bar gene (A – genotype Pt, B – genotype f2). M – marker; K- – water; K+ – pBIBar; Pt, f2 – non-transgenic control; 1 – PtXIBar4a; 2 – PtXIBar9a; 3 – PtXIBar14a; 4 – PtXIBar23a; 5 – PtXIBar29a; 6 – PtXIBar30a; 7 – PtXIBar31a; 8 – f2XIBar1a; 9 – f2XIBar2a; 10 – f2XIBar3a; 11 – f2XIBar4a; 12 – f2XIBar5a; 13 – f2XIBar6a; 14 – f2XIBar8a

Genetic transformation with the pBIBar vector produced eighteen kanamycin-resistant aspen lines: ten lines of the Pt genotype and eight lines of the f2 genotype. DNA for PCR analysis was isolated from the 14 best *in vitro *growing lines (seven of each genotype). PCR analysis of the *virB *gene revealed no agrobacterial contamination of DNA samples. The presence of a *nptII *selective gene sequence was confirmed in all lines (data not shown). Insertion of the target *bar *gene into the aspen genome was detected in six out of seven Pt lines (except for PtXIBar23a); all f2 lines contained amplification of the DNA fragment of the expected size (310 bp, *Fig. 1*).

**Fig. 2:**
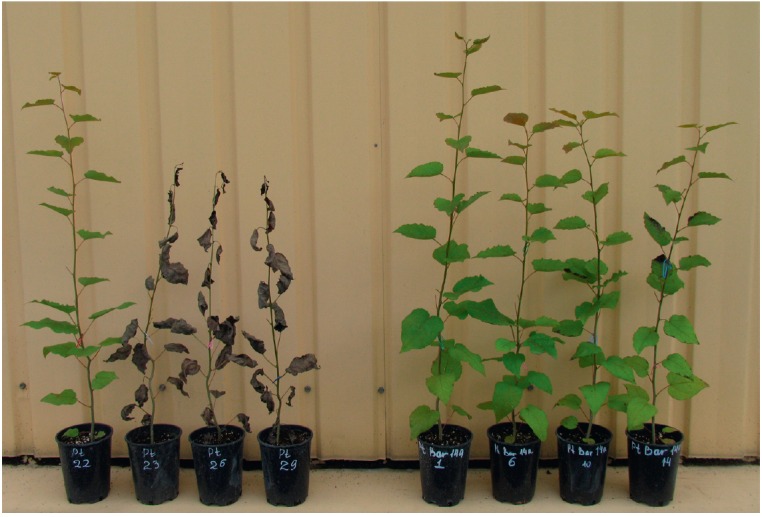
One-year control and transgenic aspen plants (genotype Pt) 7 days after treatment with water or the Basta herbicide at doses of 2.5, 5, 10 l/ha in 2014. Left – untransformed control plant, right – transgenic line PtXIBar14a

*In vitro *resistance of aspen plants (13 lines and two source genotypes) was determined by rooting in a medium containing 0 (control), 0.5 mg/l (sublethal concentration), or 5 mg/l (lethal concentration) of PPT. Two weeks after planting, the non-transgenic plants in the medium with 0.5 mg/l PPT displayed a dramatically decreased rate of rooting, number and length of roots, whereas all non-transgenic plants in the medium with 5 mg/l PPT had died. PPT treatment did not affect the rate of rooting of the transgenic plants, although some lines had a lower number and shorter length of roots. Three transgenic lines of each genotype were selected based on the results of the *in vitro *experiment: PtXIBar9a, PtXIBar14a, PtXIBar29a, f2XIBar2a, f2XIBar3a, and f2XIBar5a, all of which displayed no decrease in rooting parameters in the medium with PPT. These lines were evaluated for resistance to Basta herbicide outdoors. The one-year-old non-transgenic aspen plants displayed low resistance: within 3 days all leaves on plants of both genotypes were completely necrotic, regardless of the herbicide doses used (*Table 1* and *Table 2*). All transgenic lines were resistant to treatment with 2.5 l/ha of the herbicide, and two lines of the f2 genotype were also resistant to a dose of 5 l/ha. In the remaining cases, some leaves had small spots of necrosis, up to 5–10% of leaf area. Within 7 days after treatment with Basta at the maximum dose, the degree of damage had increased in some transgenic lines: a higher number of affected leaves and larger necrosis area (up to 25% of the leaf area). 14 and 28 days after the treatment there was no further progression of damage in the transgenic plants, while all leaves fell from the control plants that died. The appearance of the plants 7 days after the treatment is shown in *Fig. 2*.

**Fig. 3:**
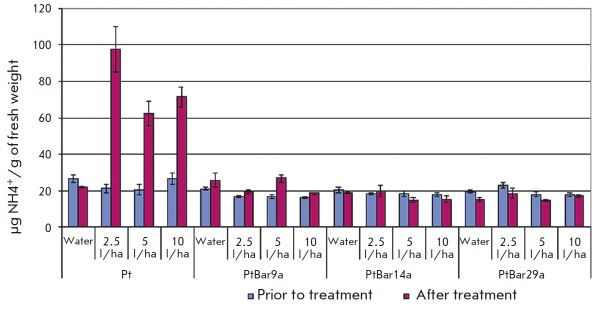
Foliar ammonia concentrations in leaves of aspen plants (genotype Pt) before and 3 days after Basta herbicide treatment

**Fig. 4:**
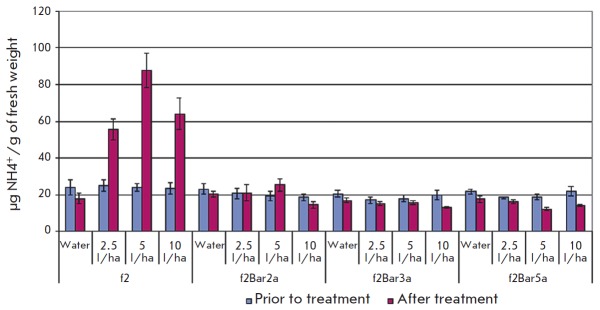
Foliar ammonia concentrations in leaves of aspen plants (genotype f2) before and 3 days after Basta herbicide treatment

Foliar NH4-N concentrations were similar in all oneyear- old transgenic aspen plant lines, while the ammonium content in the control plants was significantly higher: 17.5–19.6 and 24.2 •µg NH4+/g of fresh weight for the Pt genotype (*p * < 0.001) and 18.9–20.6 and 24.1 •µg NH4^+^/g of fresh weight for the f2 genotype (*p* < 0.05), respectively. Three days after the treatment, the ammonium concentration in the control plants had increased in a dose-dependent manner: 2.7–4.6-fold for the Pt genotype (*Fig. 3*) and 2.2–3.7-fold for the f2 genotype (*Fig. 4*). In most transgenic lines, the ammonium concentration had decreased (up to –36% from the baseline); however, for all versions of the PtXIBar9a line its concentration had increased by 14–60% (with no significant difference compared to water treatment in absolute terms).

**Table 1:** Resistance of aspen plants of the Pt genotype to Basta herbicide treatment

Line	Herbicide dose, l/ha	Degree of necrosis, points	
		After 3 days	After 7 and 14 days
Pt	2.5	5	5
	5	5	5
	10	5	5
PtXIBar9a	2.5	0	0
	5	< 1^*^	< 1
	10	< 1	1^**^
PtXIBar14a	2.5	0	0
	5	< 1	< 1
	10	1	1
PtXIBar29a	2.5	0	0
	5	< 1	< 1
	10	< 1	1

^*^Up to 1/3 of all leaves were affected (necrosis up to 5–10% of the total area).

^**^Up to 1/2–2/3 of all leaves were affected (necrosis up to 25% of the total area).

Prior to the treatment, aspen leaves contained, depending on the line, 55.9–64.1% of water (*Table 3*). Herbicide treatment caused sharp dehydration in the control plants: water content dropped to 20.3–24.0% for plants with the Pt genotype and to 22.7–25.3% for plants with the f2 genotype. There was almost no change in this parameter in the transgenic plants, with post-treatment values of 53.9–63.3% (95–102% of the baseline). There were no significant differences between various herbicide treatment options.

**Table 2:** Resistance of aspen plants of the f2 genotype to Basta herbicide treatment

Line	Herbicide dose, l/ha	Degree of necrosis, points	
		After 3 days	After 7 and 14 days
f2	2.5	5	5
	5	5	5
	10	5	5
f2XIBar2a	2.5	0	0
	5	0	0
	10	< 1	1
f2XIBar3a	2.5	0	0
	5	0	< 1
	10	< 1	1
f2XIBar5a	2.5	0	0
	5	< 1	< 1
	10	< 1	< 1

**Table 3:** Water content in aspen leaves before and after Basta herbicide treatment

Genotype	Line	Treatment	Water content, %	
			Prior to treatment	After treatment
Pt	Pt	Water	64.0	61.6
		Herbicide	61.3–64.1	20.3–24.0
	PtXIBar9a	Water	59.5	55.7
		Herbicide	56.0–60.4	53.9–57.2
	PtXIBar14a	Water	59.8	56.7
		Herbicide	60.5–62.1	59.5–60.8
	PtXIBar29a	Water	59.7	59.2
		Herbicide	57.4–61.7	56.9–61.0
f2	f2	Water	55.9	52.6
		Herbicide	59.1–61.5	22.7–25.3
	f2XIBar2a	Water	60.6	59.5
		Herbicide	60.9–61.6	58.7–61.3
	f2XIBar3a	Water	60.8	61.1
		Herbicide	60.6–62.9	60.7–63.3
	f2XIBar5a	Water	59.9	59.0
		Herbicide	62.4–63.2	60.4–61.6

**Fig. 5:**
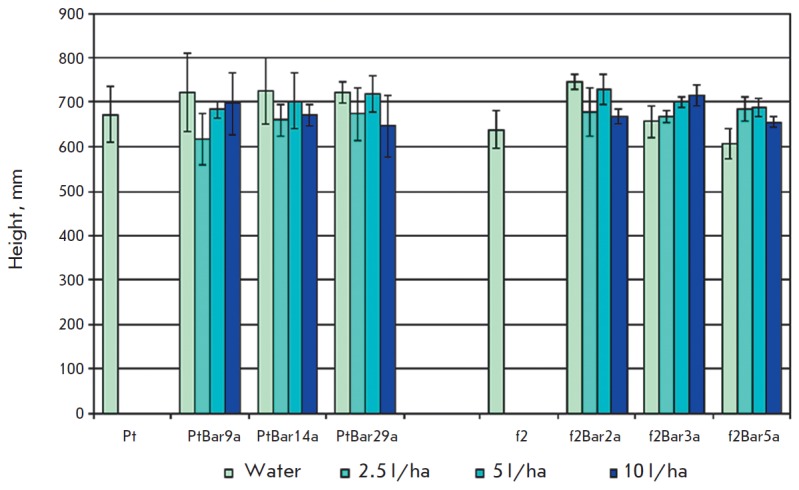
Effect of Basta herbicide treatment on the growth of control and transgenic aspen plants in 2014

Measurements of biometric parameters of the aspen plants in the 2014 season did not reveal any negative impact of the herbicide treatment on the growth of the transgenic lines. There were no statistically significant differences between various treatments in plant height as measured at the end of the growing season (*Fig. 5*). The transgenic lines also did not differ in height from each other or from the control plants. There were no significant differences in foliage (data not shown), but the basal diameter of the f2XIBar5a plant line treated with 2.5 or 5 l/ha was significantly higher than in the same line treated with water: 6.9, 7.0, and 6.3 mm, respectively (*p * < 0.05).

**Fig. 6:**
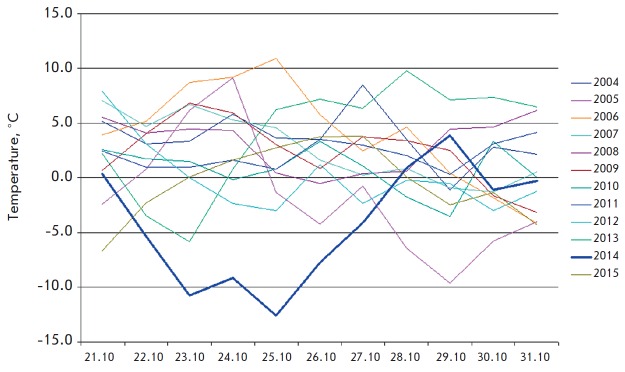
Minimum temperature in 21-31 October for 2004-2015 in Pushchino

To assess the impact of abiotic factors on the stability of the transferred gene expression, the plants were subjected to overwintering outdoors. In late October 2014, anomalously low temperatures were observed throughout the European part of Russia. In Pushchino, the temperature dropped to -12.6 °C, which is ca. 10 °C lower than average long-term values (*Fig. 6*).

**Fig. 7:**
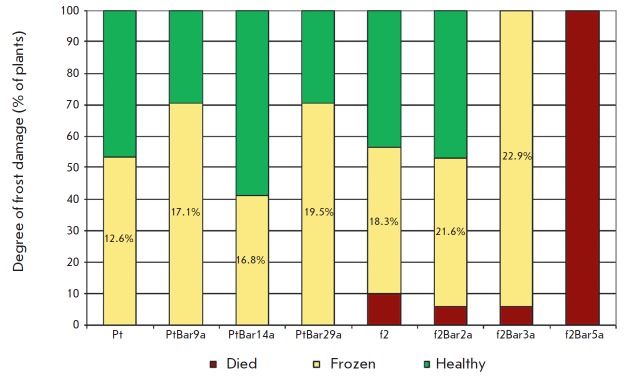
Damage of aspen plants after overwintering

In spring, after breaking of buds it was discovered that this frost caused partial freezing of one-year-old shoots and even plant death (*Fig. 7*). The f2 genotype displayed significantly lower freeze tolerance than the Pt genotype. All plants of the f2XIBar5a line had died; all shoots of the f2XIBar3a line were partially frost-damaged (on average 22.9% of their length were affected) and only in line f2XIBar2a and in the controls did roughly one half of the plants sustain no damage. None of the Pt genotype plants had died, and the share of those was 41.2–70.6% with a lower degree of shoot frost damage.

**Fig. 8:**
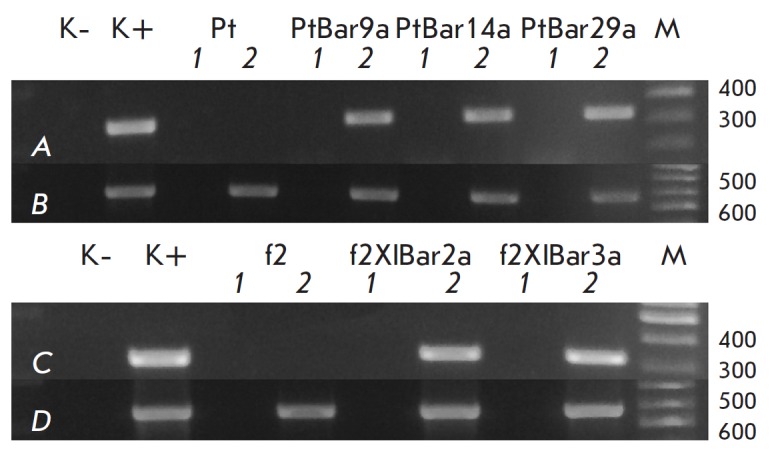
RT-PCR analysis of the expression of the bar gene (A, C) in transgenic aspen plants (A, B – genotype Pt, C, D – genotype f2). Actin was used as an internal control (B, D). K- – water; K+ – DNA non-transgenic plants (actin) or pBIBar (bar); 1 – RNA from transgenic lines; 2 – cDNA from transgenic lines; M – marker

RT-PCR analysis of total RNA of the five surviving aspen transgenic lines revealed a positive signal of the expected size for all lines, confirming the transcription of the *bar *gene (*Fig. 8*). There was no transcription of the *bar *gene in non-transgenic plants of both genotypes.

**Fig. 9:**
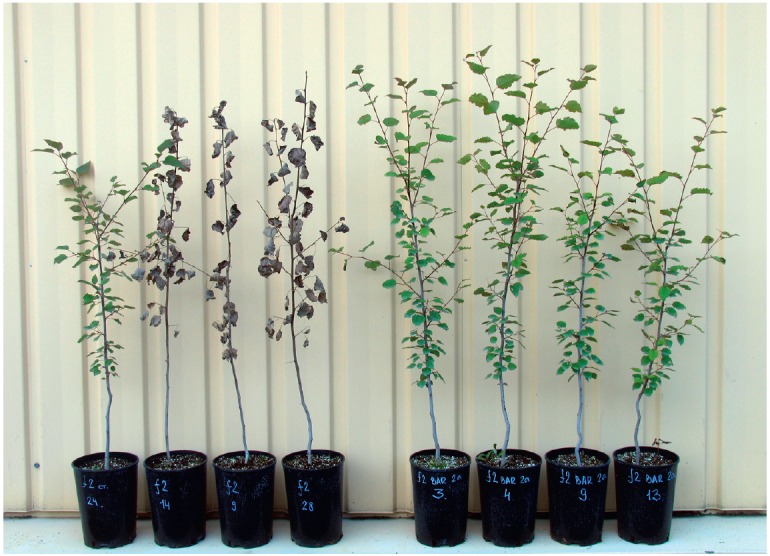
Two-year control and transgenic aspen plants (genotype f2) 7 days after treatment with water or the Basta herbicide at doses of 2.5, 5, 10 l/ha in 2015. Left – untransformed control plant, right – transgenic line f2XIBar2a

In 2015, the surviving plants of the five transgenic aspen lines and the initial genotypes were re-treated with Basta herbicide (the lost control plants had been replaced with the reserve). The onset of damage signs in two-year-old non-transgenic plants had slightly slowed down compared to the one-year-old ones back in 2014: three days after the treatment at doses equivalent to 2.5 and 5 l/ha, the leaves still had living tissue sections (four-point damage). However, 7 days after the treatment all the leaves of non-transgenic plants were completely necrotic (*Fig. 9*). There were no significant differences in resistance among the transgenic lines. All plants were fully resistant to the 2.5 l/ha dose. Treatment with 5 l/ha did not cause any damage within 3 days: within 7 days, small necrosis spots (up to 1 mm) had appeared on some leaves, and within 14 days approximately 25% of all of the leaves displayed signs of damage in the form of necrotic spots or strips along the edges of a leaf no more than 1 mm in width. The effect of treatment with a twofold normal field dosage (10 l/ha) was more pronounced: small spots of necrosis on single leaves were detected already on Day 3 after the treatment, and within 7 days up to a third of all leaves were affected, with the number of affected leaves increasing to nearly half after 14 days. For this treatment, small spots of necrosis (1–2 mm in diameter) were observed primarily on the edges of the leaves, and only in some leaves at the top of the shoots (2–3 leaves per plant) had the necrosis affected 10–15% of the area. 14 days after the treatment, there was no further progression of the damage.

Four transgenic lines PtXIBar9a, PtXIBar14a, PtXIBar29a, and f2XIBar2a were selected based on the results of the study as highly resistant to PPT herbicide with a maximum level of freeze tolerance.

## DISCUSSION

Modern forest plantations are intensively managed artificial forests for wood production with a level of efficiency much higher than the productivity of natural forests. There are several ways to achieve this: e.g. the use of elite genotypes, including transgenic ones. For example, in April 2015 Brazil approved commercial use of transgenic eucalyptus with accelerated growth [24]. An equally important issue is the quality of management and the use of high-quality planting material, whose cultivation in nurseries is impossible without weed control. A chemical method of weed control can increase the yield of planting material and improve its quality, while simultaneously significantly reducing both labor and financial costs. Imparting resistance to herbicides by genetic engineering methods simplifies the implementation of a chemical method of weed control without damaging the cultivated plants. For this purpose, the *bar *gene from soil bacterium *S. hygroscopicus *[9] was inserted into aspen plants. In addition to conferring resistance to PPT-based herbicides, this gene is also one of the most widely used selective genes in genetic engineering [25]. Moreover, unlike most other herbicide-resistance genes, the *bar* gene ensures inactivation of the active ingredient of a herbicide.

Elite aspen genotypes of Russian origin, characterized by rapid growth and resistance to trunk rot, were used for the transformation. For example, the plantings of clone 34 (the source material for the *in vitro* culture of the f2 genotype) at the age of 47 years exceeded plantings of common aspen in the sum of basal area by 51%, in stock by 43%, while the share of trees with trunk rot in this clone was 4.7 times lower [26]. PCR confirmed *bar *gene insertion in 13 transformants. An *in vitro *experiment demonstrated the resistance of all transgenic lines to PPT concentration in the medium, which is lethal for non-transgenic plants, confirming expression of the inserted gene. Further tests of aspen plants resistance to herbicides were carried out under semi-natural conditions: the growth of the root system was limited by the volume of the planting container, but the plants were kept outdoors and were exposed to all effects of the environment. Currently, it is the closest possible approximation of natural conditions for transgenic plants available in Russia, since field tests have not been performed for approximately 10 years. The plants were treated with water or Basta herbicide at doses equivalent to 2.5, 5, and 10 l/ha. This herbicide is used as a desiccant at a dose of 1.5–2.5 l/ ha and as an herbicide at a dose of 4–5 l/ha. Therefore, the maximum concentration was equivalent to twofold normal field dosage. To assess the consistency of the transferred trait, the treatment was performed in 2014 and 2015, after overwintering outdoors.

Treatment of one-year-old plants demonstrated that aspen is very sensitive to PPT: 3 days after the treatment, all leaves of the untransformed controls were completely necrotic. The high sensitivity of plants of the genus *Populus *to PPT has been reported previously: complete necrosis of *P. alba *leaves was observed as early as 2 days after treatment with a standard field dosage of the herbicide [3]. In contrast to the controls, transgenic aspen plants carrying the *bar *gene demonstrated a high degree of resistance: treatment with 5 and 10 l/ha doses resulted in only small spots of necrosis. The herbicide did not cause retardation of growth in any of the six aspen lines, whereas Meilan *et al*. [6] observed a decrease in the growth of 25% of *Populus* hybrid lines treated with a single dose of the herbicide and in 17–61% of plants treated with a double dose. Other trees with the *bar *gene also exhibited a high degree of resistance: eucalyptus [11] and *P. alba *[3] plants proved resistant to a double dose of PPT herbicides. We have observed differences between the genotypes in their response to herbicide treatment: signs of damage were more pronounced in transgenic lines of the Pt genotype than in those of the f2 genotype. Our *P. tremula* plants that died after treatment with 375 g/ha PPT proved to be more sensitive to PPT than the *P. alba *×* P. tremula *hybrid, which survived after being treated with 400 g/ha PPT [10].

In plant cells, the ammonium, released after nitrate reduction, amino acid degradation and photorespiration, can only be effectively detoxified by glutamine synthetase [7]; therefore, plants are highly sensitive to inhibitors of this enzyme, including PPT. The accumulation of ammonium in PPT-treated plants is widely used as a biochemical marker of glutamine synthetase inhibition [27]. Three days after treatment, the ammonium concentration in one-year-old non-transgenic aspen plants increased 2.2–4.6-fold depending on both the dose and the genotype. Apparently, the Pt genotype is more sensitive to the action of the herbicide (2.7–4.6-fold increase) than f2 (2.2–3.7-fold increase). The observed increase in ammonium concentration in aspen was far less pronounced than in the *P. alba* × *P. tremula *hybrid, for which within 24 hours after the treatment there was nearly a 100-fold increase in ammonium content: from 9 to 800–900 •µg/g of fresh weight [10]. This can be attributed to the differences in genotype, time after treatment (72 and 24 hours), dose of PPT (0.375–1.5 and 4 kg/ha), and metabolic rate associated with the age of the plant, as well as to being in a greenhouse or outdoors. In all six lines of transgenic aspen, treatment with the herbicide did not cause any significant increase in ammonium concentration, which was quite similar in all cases (12.4–27.0 •µg/g of fresh weight). In this respect, our results differ from the data by Asano et al. [28], who observed an approximately 10-fold variation in the ammonium concentration in six transgenic *Agrostis *lines carrying the *bar* gene, which almost reached the level of non-transgenic plants 3 days after treatment. In contrast, we observed a decrease in ammonium concentration in most lines and in three cases this decrease reached 34–36%, which was statistically significant (f2XIBar3a after 10 l/ha treatment, f2XIBar5a and after 5 and 10 l/h treatment). This may be associated with some processes occurring within this 3-day period, such as incorporation of ammonium into nitrogen metabolism. High doses of the herbicide that caused necrosis of leaves in the transgenic plants did not affect the ammonium content.

The toxicity of the accumulated ammonium is considered to be the major factor of PPT herbicidal activity [8, 29]. On the other hand, it has been demonstrated that the action of the herbicide is primarily due not to the accumulation of ammonia, but to the lack of glutamine, which makes it impossible to synthesize important nitrogen-containing compounds that are normally produced from glutamine amide and amine nitrogen [30]. Complete necrosis of the non-transgenic aspen plants leaves that occurs only at a 2.2-fold increase in ammonium levels, the lack of correlation between leaves damage and levels of ammonium in the transgenic plants, as well as the plants ability to survive despite a manifold increase in ammonium levels [31] taken together suggest that ammonium phytotoxicity is not the primary cause of aspen plants death after PPT treatment.

To assess PPT sensitivity, we used such an indicator as the decrease in fresh [8] or dry [32] weight. Since Basta herbicide is used also as a desiccant, we decided to use the rate of dehydration of leaf tissue. The herbicide caused sharp dehydration in non-transgenic plants: there was an almost 3-fold drop in water content, regardless of the dose. Apparently, already a dose of 2.5 l/ha (the standard dose for desiccation) is enough for a plant to reach a certain physiological limit of dehydration. The appearance of necrotic spots on the leaves of transgenic plants after treatment with doses of 5 and 10 l/ha of the herbicide had no effect on their water content, which was within 95–102% of the baseline values for all plants, including those treated with water. It should be noted that there was a smaller decrease in the water content of control plants of the f2 genotype (2.4–2.7-fold) than in Pt plants (2.6–3-fold).

In mid- to late October 2014, there was a sharp drop in temperature throughout the European part of Russia, which was observed for the first time since 1982 [33]. Negative anomalies during this period reached 8–11°C, and the temperature corresponded to a mid-December one. These unplanned tests for freeze tolerance resulted in the death of all plants of the f2XIBar5a line and freezing of all plants of the f2XIBar3a line. This suggests that these lines have changes that significantly reduce their resistance to low temperatures in autumn. Interestingly, of the three transgenic lines of the f2 genotype, the two most affected ones also exhibited a significant decrease in ammonium levels after treatment with the herbicide. Freeze tolerance of the other four transgenic aspen lines was considerably higher and remained at the level of non-transgenic plants of both genotypes. This case once again confirms the need to conduct field trials of perennial plants for long periods of time and in different climatic zones.

In addition to the level of expression of the inserted genes, it is also important to test trees for the stability of its expression as trees keep growing for many years, and each year they are subjected to periods of dormancy and growth, as well as to various abiotic and biotic stresses. Unstable expression of the transferred genes and, as a consequence, unstable manifestation of new traits undermines the commercial value of such plants. Stable expression of the *bar *gene in hybrid *Populus *plants without silencing was demonstrated in a field over the course of three [34] or eight years [35]. A high level of resistance to the Basta herbicide was also observed in pears rootstock with the* bar *gene during the 5th year of cultivation in a field [36]. However, field testing of poplar with glyphosate resistance genes over the course of two years revealed a strong increase in damage in the second year in two lines out of 80 that were treated with herbicides, and some lines exhibited morphological changes [6]. In our work, strong abiotic stress did not cause a decrease in the *bar *gene expression in the surviving aspen plants, which was confirmed by RT-PCR analysis. Two-yearold transgenic plants retained a high level of resistance in the second year; however, development of signs of damage had been slowed down in all plants, including the non-transgenic controls. This can be attributed to a significant increase in leaf surface or to lower susceptibility to the herbicide due to a more developed cuticle. A less developed cuticle was used as an explanation for the decreased resistance of *Populus *hybrids with the *bar *gene, which had been treated soon after field-planting: however, 8 years later these plants displayed high resistance [35]. This version is supported by the fact that, in contrast to the first year when necrotic spots were relatively evenly distributed over the surface of the leaves, in the second year the signs of damage were concentrated on leaf edges, which could have had a thinner cuticle. It is also possible that the applied herbicide trickled to leaf edges.

Since herbicide resistance is important primarily in the first few years of tree growth, it is expedient to insert these genes into already transgenic plants. For example, the first re-transformation of woody plants was performed by insertion of the *bar *gene into transgenic pear plants already carrying the *gus *gene [37]. The potential of this approach in forest biotechnology is confirmed by research by ArborGen company (USA) in which herbicide resistance genes were transferred into a transgenic eucalyptus line AGEH427 [38] which already contained genes for freeze tolerance and sterility [39].

## CONCLUSION

Several transgenic aspen lines carrying the *bar *gene conferring resistance to herbicides containing phosphinothricin were produced from elite aspen genotypes. Two years of testing under semi-natural conditions have demonstrated resistance of the transgenic lines to a twofold normal field dosage of the Basta herbicide. Based on the results of these tests, four lines which displayed both freeze tolerance under extremely low temperatures and high resistance to herbicides (PtXIBar9a, PtXIBar14a, PtXIBar29a, f2XIBar2a) were selected. These plants are promising for further studies, in particular to field testing. In addition, the *bar *gene could be used for retransformation of the transgenic woody plants that have been obtained in our laboratory and have already demonstrated valuable traits, such as increased productivity and modification of the composition of wood [40].

## References

[1] Baum S., Weih M., Busch G., Kroiher F., Bolte A. (2009). Landbauforschung..

[2] Bubnov A.A. (2014). Proceedings of the Saint Petersburg Forestry Research Institute. (in Russian)..

[3] Confalonieri M., Belenghi B., Balestrazzi A., Negri S., Facciotto G., Schenone G., Delledonne M. (2000). Plant Cell Rep..

[4] Fillatti J.J., Sellmer J., McCown B., Haissig B., Comai L. (1987). Mol. Gen. Genet..

[5] Brasileiro A.C.M., Tourneur C., Leple J.C., Combes V., Jouanin L. (1992). Transgenic Res..

[6] Meilan R., Han K.H., Ma C., DiFazio S.P., Eaton J.A., Hoien E.A., Stanton B.J., Crockett R.P., Taylor M.L., James R.R. (2002). Can. J. For. Res..

[7] Miflin B.J., Lea P.J. (1977). Annu. Rev. Plant Physiol..

[8] Tachibana K., Watanabe T., Sekizawa Y., Takematsu T. (1986). J. Pestic. Sci..

[9] De Block M., Botterman J., Vandewiele M., Dockx J., Thoen C., Gossel V., Movva N.R., Thompson C., van Montagu M., Leemans J. (1987). EMBO J..

[10] De Block M. (1990). Plant Physiol..

[11] Harcourt R.L., Kyozuka J., Floyd R.B., Bateman K.S., Tanaka H., Decroocq V., Llewellyn D.J., Zhu X., Peacock W.J., Dennis E.S. (2000). Mol. Breed..

[12] González E., Gugliermoni C., Galvão M., Fagundes M., Ferreira M., Almeida G., Alves H., Gonsalves J., Silva F., Bentivenha S. (2011). BMC Proc..

[13] Alvarez R., Alvarez J.M., Humara J.M., Revilla A., Ordas R.J. (2009). Biotechnol. Lett..

[14] Bishop-Hurley S.L., Zabkiewicz R.J., Grace L., Gardner R.C., Wagner A., Walter C. (2001). Plant Cell Rep..

[15] Parasharami V.A., Naik V.B., von Arnold S., Nadgauda R.S., Clapham D.H. (2006). Plant Cell Rep..

[16] Zhigunov A.V., Shabunin D.A., Butenko O.Yu. (2014). Vestnik of Volga State University of Technology. Series “Forest. Ecology. Nature Management.”. (in Russian)..

[17] Lloyd G., McCown B. (1981). Proc. Int. Plant Prop. Soc..

[18] Padegimas L., Shulga O.A., Skryabin K.G. (2004). Molecular Biology. (in Russian)..

[19] Lebedev V.G., Schestibratov K.A., Shadrina T.E., Bulatova I.V., Abramochkin D.G., Miroshnikov A.I. (2010). Russian Journal of Genetics. (in Russian)..

[20] Rogers S.O., Bendich A.J. Plant Mol. Biol. Manual. Kluwer Acad..

[21] De Block M., De Brouwer D., Tenning P. (1989). Plant Physiol..

[22] Weatherburn M. (1967). Anal. Chem..

[23] Chang S., Puryear J., Cairney J.A. (1993). Plant Mol. Biol. Rep..

[24] (2015). Nat. Biotechnol..

[25] Green J.M., Micheal D.K. (2011). J. Agric. Food Chem..

[26] Bagaev S.N., Bagaev E.S. (1990). Forestry. (in Russian)..

[27] Avila-Garcia W.V., Carol Mallory-Smith C. (2011). Weed Sci..

[28] Asano Y., Ito Y., Fukami M., Sugiura K., Fujiie A. (1998). Plant Cell Rep..

[29] Pornprom T., Chompoo J., Grace B. (2003). Weed Biol. Management..

[30] Evstigneeva Z.G., Solov’eva N.A., Sidel’nikova L.I. (2003). Applied Biochemistry and Microbiology. (in Russian)..

[31] Petrović A., Yoshida Y., Ohmori T. (2009). J. Hort. Sci. Biotech..

[32] Nolte S.A., Young B.G., Mungur R., Lightfoot D.A. (2004). Weed Res..

[33] (2014). Hydrometeorological Centre of Russia, 03.11.2014.

[34] Li J., Brunner A.M., Meilan R., Strauss S.H. (2009). Tree Physiol..

[35] Li J., Meilan R., Ma C., Barish M., Strauss S.H. (2008). West J. Appl. For..

[36] Lebedev V.G., Dolgov S.V. (2008). Acta Hort..

[37] Lebedev V.G., Skryabin K.G., Dolgov S.V. (2002). Acta Hort..

[38] Gulledge E., Judy C., Cunningham M. AAIC 25th Anniversary Meeting, October 12–16, 2013, Washington, USA..

[39] Zhang C., Norris-Caneda K.H., Rottmann W.H., Gulledge J.E., Chang S., Kwan B.Y., Thomas A.M., Mandel L.C., Kothera R.T., Victor A.D. (2012). Plant Physiol..

[40] Shestibratov K., Lebedev V., Podrezov A., Salmova M. (2011). BMC Proc..

